# Epidemiology of multidrug resistant bacterial organisms and *Clostridium difficile* in German hospitals in 2014: Results from a nationwide one-day point prevalence of 329 German hospitals

**DOI:** 10.1186/s12879-016-1756-z

**Published:** 2016-09-02

**Authors:** Nils-Olaf Huebner, Kathleen Dittmann, Vivien Henck, Christian Wegner, Axel Kramer, for the Action Group Infection Prevention (AGIP)

**Affiliations:** 1 Institute of Hygiene and Environmental Medicine, University Medicine Greifswald, Walther-Rathenau-Str. 49a, 17475 Greifswald, Germany; 2 IMD laboratory network, MVZ Greifswald GmbH, 17489 Greifswald, Germany

**Keywords:** Point prevalence, MDROs, HICARE-network, MRSA, CD, ESBL, VRE, Infection control staff, Type of screening

## Abstract

**Background:**

One important aspect in combatting resistance to antibiotics is to increase the awareness and knowledge by epidemiological studies. We therefore conducted a German-wide point-prevalence survey for multidrug resistant bacterial organisms (MDROs) and *Clostridium difficile* (CD) to assess the epidemiology and structure quality of infection control in German hospitals.

**Method:**

1550 hospitals were asked to participate and to report surveillance data on the prevalence of Methicillin-resistant and Vancomycin resistant *Staphylococcus aureus* (MRSA, VRSA/GRSA), Vancomycin resistant *Enterococcus faecalis/faecium* (VRE), multiresistant strains of *Escherichia coli* (EC), *Klebsiella spp.* (KS), *Enterobacter spp*. (ES), *Acinetobacter spp*. (AB) and *Pseudomonas spp. (PS).* as well as CD infections.

**Results:**

Surveys from 73,983 patients from 329 hospitals were eligible for analysis. MRSA was the most often reported pathogen (prevalence: 1.64 % [CI95: 1.46-1.82]), followed by 3 multidrug resistant EC (3MRGN-EC) (0.75 % [CI95: 0.60–0.89]), CD (0.74 % [CI95: 0.60–0.88]), VRE (0.25 % [CI95: 0.13–0.37]) und 3MRGN-KS (0.22 % [CI95: [0.15–0.29]). The majority of hospitals met the German recommendations for staffing with infection control personnel.

**Conclusion:**

The continuing increase in participating hospitals in this third survey in a row indicates a growing awareness to MDROs and our pragmatic approach. Our results confirm that MRSA, 3MRGN-EC, VRE and 3MRGN-KS remain the most prevalent MDROs in German hospitals.

## Background

Antibiotic resistance of bacterial pathogens is an emerging problem worldwide [1]. While no longer limited to in-patient care, hospitals are still a focal point for transmissions and infections with multidrug resistant organisms (MDRO). While for decades Methicillin-resistant *S. aureus* (MRSA) strains was the leading MDRO, the raise in antibiotic resistance in Gram-negative bacteria and of other nosocomial pathogens like *Clostridium difficile* (CD) have aggravated the problem of antibiotic resistance and let to increased efforts to control MDROs [2].

One important aspect in combatting MDROs is to increase awareness to as well as knowledge on the epidemiology of MDROs. For Germany, a number of regional surveys have been conducted by so called regional MDRO-networks of care providers the last years. These networks have now been established in all states in Germany based on a resolution of the Conference of Ministers of Health ("Gesundheitsministerkonferenz") in 2006 [3]. Most of these surveys were based on an admission screening and covered MRSA only [4–8].

In a complementary approach, the German Society of Hospital Hygiene conducted a first German-wide point-prevalence survey for MDROs to assess the in-house epidemiology in German hospitals in 2010. This survey assessed the epidemiology of MRSA and other MDROs by gathering routine data by a questionnaire [9]. Two years later, the survey was repeated as collaboration between the then newly founded Action Group Infection Prevention, an interdisciplinary expert group, with the “HICARE-Health Region Baltic Sea Coast”, a project supported by the Federal Ministry of Education and Research as part of the German strategy against bacterial resistances (Deutsche Antibiotika-Resistenzstrategie, DART) [10, 11]. While the fist survey included only 3,411 patients from nine hospitals, the second survey had a much higher response rate and included 13,000 patients from 56 hospitals distributed overall Germany. Here, we report the results of the third survey that was, encouraged by positive response, conducted in 2014 [9, 12].

## Method

We conducted a voluntary, anonymous, point-prevalence survey gathering routine data of structure quality in infection control and microbiological surveillance data that has to be present in hospitals in Germany by law in February 2014 [13]. The survey relied on aggregated routine data only, no informed consent from individual patients was needed [14]. The method used was approved by the Ethics Committee of the Board of Physicians Mecklenburg-West Pomerania at the University of Greifswald.

The survey consisted of three parts: one part assessed the prevalence of MDRO (calculated as proportion of the in-patients with a certain MDRO to all in-patients), one part assessed basic structure of the hospitals (e.g. data on the level of care, number of beds) and one part assessed structure quality data of infection control in the hospitals (e.g. staffing with infection control personnel, presence of admission screening for MDROs). Finally, we asked by whom and by which method the epidemiological data were provided.

To allow comparisons to the former surveys as well as to the former distinction between primary, secondary and tertiary care hospitals, only data from intensive care units, surgical and medical wards were collected.

Based on the survey forms used in 2010 [9] and 2012 [12], an updated version of former surveys was generated and converted in an active PDF-form (Adobe Acrobat X). In contrast to the former surveys that asked the epidemiology of extended-spectrum beta lactamase (ESBL) positive strains, the updated version in this survey used the “multidrug resistant Gram-negative” (MRGN)-classification of the German Commission on hospital hygiene and infection protection (KRINKO) at the Robert Koch Institute (RKI) for multi-resistance in Gram-negative bacteria [15]. This classification is meant to highlight the clinical impact of resistance of *Enterobacteriaceae*, *Pseudomonas spp.* and *Acinetobacter spp.* and is based on the sensitivity against four important classes of antimicrobials: acylureidopenicillins, 3rd and 4th generation cephalosporins, quinolones and carbapenems. According to this classification a multidrug resistant strain is classified as either 3MRGN (resistant to three out of four classes) or 4MRGN (resistant to four out of four classes) [15].

The following bacterial pathogens were included in the survey: *Methicillin-resistant Staphylococcus aureus* (MRSA), *Vancomycin resistant S. aureus* (VRSA/GRSA), *Vancomycin resistant Enterococcus faecalis*/ *E. faecium* (VRE), 3MRGN and 4MRGN *Escherichia (E.) coli* (3MRGN-EC; 4MRGN-EC), 3MRGN and 4MRGN *Klebsiella spp.* (3MRGN-KS; 4MRGN-KS), 3MRGN and 4MRGN *Enterobacter spp.* (3MRGN-EB; 4MRGN-EB), 3MRGN and 4MRGN *Acinetobacter spp.* (3MRGN-AB; 4MRGN-AB), 3MRGN and 4MRGN *Pseudomonas spp.* (3MRGN-PA; 4MRGN-PA) as well as *Clostridium difficile* (CD) infections with infections in intensive care unit (ICU)-patients or requiring ICU-treatment as sub-group. We asked for *Clostridium difficile* colonisations, because we knew, that CDI patients, who were no longer suffering from diarrhoea but still in isolation were not well reported under “infections” to assess this group too. Hospitals were asked whether an outbreak with these pathogens was ongoing at the day of the survey to control for this possible confounder.

The participants were asked to report the prevalence rates as known by health care workers and infection control personnel in the hospitals. We therefore deliberately gave no generic definition for cases or to distinguish between infections/colonisations and nosocomial and hospital acquired cases, respectively. Instead, we aimed to collect the routine data exactly as used for clinical and infection control decisions (e.g. isolation measures), for reimbursement and for continuous recording of the occurrence of MDROs that has is required in all hospitals in Germany by law (IFSG). Case definitions were therefore based on microbiological results as well as information from case histories and so called “transfer sheets” (German “Überleitbögen”), which are required to share information on a present MDRO – carrier status between medical facilities.

The form was sent by E-mail to 1,550 hospitals by the last week of January 2014. Hospitals were asked to perform the survey in February 2014. Returned surveys were collected and consolidated using build-in functions of Adobe Acrobat and exported to Microsoft Excel. Microsoft Excel and IBM SPSS Statistics 22 were used for statistical analysis.

Prevalence rates were calculated on hospital/ward level and mean prevalence rates and confidence intervals were calculated as average of the prevalence rates of the former. For statistical comparisons, confidence intervals were used as robust and conservative alternative to p-values were ever possible due to the explorative nature of the survey and to avoid problems associated with multiple statistical testing explorative nature of the survey and as robust and conservative alternative to p-values [16]. Were confidence intervals implied a true difference between two groups, a t-test was performed to test for statistically significant differences when appropriate.

To calculate the percentage of patients with nosocomial infections due to MRSA, VRE, ESBL-*E.coli* and CD from the point prevalence study of the ECDC , the following formula was used [17]:

$$ \begin{array}{l} prevalence\; of\; nosocomial\; in fections\; due\;to\;a\; given\; MDRO\\ {}=\frac{nosocomial\; in fections\; with\;a\; given\; organism* percentage\; of\; resistant\; strains\; of\  that\; organism}{number\; of\; patients\; in\; the\; survey}\;\end{array} $$

## Results

### Participating hospitals

In total, 364 hospitals returned the questionnaire (return rate: 23.5 %) of which 329 (90.4 %) representing 120,180 beds were finally eligible for analysis (Table 1). Of those included, 45 (13.7 %) were tertiary care providers, 76 (23.1 %) were secondary and 208 (63.2 %) were primary care hospitals. Overall, 73,983 patients were included. The average number of beds in tertiary care, secondary care and primary care providers was 800, 400 and 259, respectively.

**Table 1:** Number, beds, patients and compliance of staffing included in 329 hospitals and divided by level of care (tertiary, secondary and primary)

Level of care	Number (n)	Beds (n)	Patients (n)	Hospitals that comply with the requirements for staffing for ICN (n, %)	Hospitals that comply with the requirements for staffing for ICC (n, %)
Tertiary	45	36,001	20,143	37 (82.2 %)	36 (80.0 %)
Secondary	76	30,367	18,341	64 (84.2 %)	53 (69.7 %)
Primary	208	53,812	35,499	178 (85.6 %)	157 (75.5 %)
Total	329	120,180	73,983	279 (84.8 %)	246 (74.8 %)

(ICN = infection control nurse, ICC = infection control consultant)

**Fig. 1:**
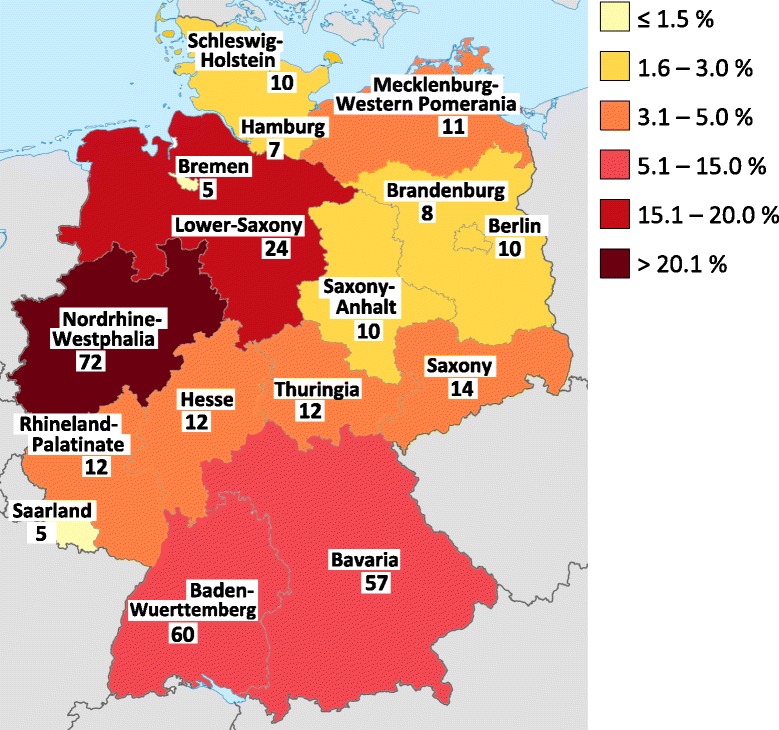
Numbers and percentages (depicted by different colours) of participating hospitals by state (modified from Wikimedia Commons Germany Location Map by NordNordWest, licensed under CC-sa) [24]

Figure 1 shows the participation of hospitals divided by federal German states. In the majority of states between 15 – 25 % of hospitals could be included in the study. Bremen, Mecklenburg-Western Pomerania, Thuringia and Baden-Wuerttemberg showed the highest participation, while Hesse was the only state with participation below 10 %. In total, hospitals from Northrhine-Westphalia made up the larges fraction of participating hospitals (21.3 %), followed by Baden-Wuerttemberg (18.2 %) and Bavaria (17.3 %) and Mecklenburg Western Pomerania (7.3 %).

### Staffing with infection control personnel

The survey revealed that the German recommendations by KRINKO for staffing with infection control personnel were not fully met by the participating hospitals regardless of the level of care (Table 1) [18]. In total, 279 of 329 (84.8 %) hospitals met the staffing needs for infection control nurses and 246 (74.8 %) hospitals for infection control consultants (Table 1).

### MDRO-Screening policy

In total, 89.1 % of included hospitals reported to have some kind of MRSA-admission screening, with marginal differences between levels of care (tertiary 91.1 %, secondary 88.2 %, primary 88.9 %) (Table 2). Most screening regimes (79.3 %) followed the recommendation of KRINKO to screen defined groups of risk patients [19]. Another 6.7 % reported to have a universal admission screening and 3.0 % did only screen admissions to critical wards like ICUs or transplant units (Table 2).

**Table 2:** MRDO-Screening strategy

	MRSA				4MRGN	VRE	ESBL
	Universal screening *	Risk-based **	Only admission to critical wards	Not reported	Admission screening		
Tertiary (*n* = 45)	2 (4.4 %)	35 (77.8 %)	4 (8.9 %)	4 (8.9 %)	30 (66.7 %)	19 (42.2 %)	15 (33.3 %)
Secondary (*n* = 76)	6 (7.9 %)	58 (76.3 %)	3 (3.9 %)	9 (11.8 %)	34 (44.7 %)	18 (23.7 %)	19 (25.0 %)
Primary (*n* = 208)	14 (6.7 %)	168 (80.8 %)	3 (1.4 %)	23 (11.1 %)	62 (29.8 %)	32 (15.4 %)	38 (18.3 %)
total (*n* = 329)	22 (6.7 %)	261 (79.3 %)	10 (3.0 %)	36 (10.9 %)	126 (38.3 %)	69 (21.0 %)	72 (21.9 %)

* all admissions, ** according to KRINKO

Screening for other MDROs was much less common. Only 38.3 % of hospitals reported to screen for 4MRGN, 21.9 for ESBL (as proxy for 3MRGN) and 21 % for VRE. There was a marked difference in the policy to screen for MDRO other than MRSA between levels of care. Hospitals with a higher level of care were more likely to screen for MDROs other than MRSA (Table 2). The association between the screening policies for certain MDROs and the reported prevalence rates are show in Table 3 and discussed below.

**Table 3:** Prevalence rates of MDROs divided by screening strategy (non-overlapping Cis marked with *)

Screening for	MDRO	Screening			No screening			
		Number of hospitals	Mean prevalence [95 % CI]		Number of hospitals	Mean prevalence [95 % CI]		
MRSA	MRSA	324	1.64	[1.46 – 1.82]	5	0.89	[-0.95 – 2.72]	
- MRSA (all universal)		22	2.49	[1.59 – 3.40]				
- MRSA (risk based – KRINKO)		261	1.59	[1.39 – 1.79]				
- MRSA (risk based - in-house regime)		10	1.65	[0.83 – 2.47]				
- not reported		31	1.55	[0.86 – 2.24]				
ESBL	3MRGN-EC	72	1.27	[0.83 – 1.71]*	257	0.60	[0.46 – 0.73]	*
	3MRGN-KS		0.40	[0.14 – 0.66]		0.17	[0.12 – 0.22]	
	3MRGN-ES		0.24	[0.03 – 0.45]		0.06	[0.04 – 0.09]	
	3MRGN-AB		0.01	[-0.01 – 0.03]		0.02	[0.00 – 0.04]	
	3MRGN-PS		0.52	[0.04 – 0.99]		0.12	[0.08 – 0.16]	
VRE	VRE	69	0.70	[0.17 – 1.23]	260	0.13	[0.08 – 0.18]	
4MRGN	4MRGN-EC	126	0.01	[-0.00 – 0.03]	203	0.01	[-0.01 – 0.03]	
	4MRGN-KS		0.09	[-0.01 – 0.19]**		0.00	[-0.00 – 0.01]	**
	4MRGN-ES		0.02	[0.00 – 0.04]		0.00	[-0.00 – 0.01]	
	4MRGN-AB		0.03	[0.00 – 0.06]		0.02	[0.00 – 0.03]	
	4MRGN-PS		0.34	[0.05 – 0.63]		0.07	[0.03 – 0.10]	

*p < 0,001; **p = 0,068

### Responsibility of data collection

In total, data were collected directly at the wards (50.8 %) or using electronic systems in most hospitals (45.0 %), whereas the infection control nurse was responsible for collecting data in the majority of hospitals (82.4 %) (Table 4).

**Table 4:** Method and responsibility of data collection

	Method of data collection				Responsibility of Data collection					
Level of care	On the ward	By phone	Electronic system	Not reported	ICN	ICC	LPIC	Controlling/QM	Other	Not reported
Tertiary (*n* = 45)	16 (35.6 %)	0 (0.0 %)	28 (62.2 %)	1 (2.2 %)	37 (82.2 %)	3 (6.7 %)	2 (4.4 %)	0 (0 %)	3 (6.7 %)	0 (0.0 %)
Secondary (*n* = 76)	43 (56.6 %)	1 (1.3 %)	32 (42.1 %)	0 (0.0 %)	53 (69.7 %)	5 (6.6 %)	11 (14.5 %)	1 (1.3 %)	5 (6.6 %)	1 (1.3 %)
Primary (*n* = 208)	108 (51.9 %)	7 (3.4 %)	88 (42.3 %)	5 (2.4 %)	181 (87.0 %)	3 (1.4 %)	13 (6.3 %)	1 (0.5 %)	8 (3.8 %)	2 (1.0 %)
Total (*n* = 329)	167 (50.8 %)	8 (2.4 %)	148 (45.0 %)	6 (1.8 %)	271 (82.4 %)	11 (3.3 %)	26 (7.9 %)	2 (0.6 %)	16 (4.9 %)	3 (0.9 %)

(ICN = infection control nurse, ICC = infection control consultant, LPIC = link physician for infection control, QM = Quality management)

### Prevalence data

#### Overview

Notwithstanding the level of care or type of ward, MRSA was the most often reported pathogen with a prevalence of 1.64 % [CI95: 1.46-1.82] of all included patients, followed by 3MRGN-EC (0.75 % [CI95: 0.60–0.89]), CD (0.74 % [CI95: 0.60–0.88]), VRE (0.25 % [CI95: 0.13–0.37]) und 3MRGN-KS (0.22 % [CI95: [0.15–0.29]) (Tables 5 and 6).

**Table 5:** Mean prevalence of the most prevalent MDROs divided by level of care

Level of care	MRSA		VRE		3MRGN-EC		3MRGN-KS		CD	
	n	[95 % CI]*	n	[95 % CI]*	n	[95 % CI]*	n	[95 % CI]*	n	[95 % CI]*
Tertiary	331	1.75 [1.40-2.11]	128	0.64 [0.33-0.94]	202	1.13 [0.86-1.41]	74	0.32 [0.21-0.43]	189	0.90[0.68-1.12]
Secondary	271	1.61 [1.16-2.07]	42	0.44 [-0.03-0.90]	132	1.10 [0.57-1.64]	56	0.43 [0.18-0.69]	107	0.86 [0.38-1.35]
Primary	564	1.62 [1.40-1.85]	37	0.10 [0.05-0.14]	189	0.53 [0.43-0.63]	40	0.12 [0.07-0.17]	210	0.65 [0.53-0.78]
Total	1166	1.64 [1.46-1.82]	207	0.25 [0.13-0.37]	523	0.75 [0.60-0.89]	170	0.22 [0.15-0.29]	506	0.74 [0.60-0.88]

* 95 % confidence interval

**Table 6:** Mean prevalence of the most prevalent MDROs divided by type of ward

Type of ward	MRSA		3MRGN EC		CD		VRE		3MRGN-KS	
	n	Mean prevalence [95 % CI]*	n	Mean prevalence [95 % CI]*	n	Mean prevalence [95 % CI]*	n	Mean prevalence [95 % CI]*	n	Mean prevalence [95 % CI]*
Surgical	429	1.22 [1.04-1.40]	177	0,46 [0.37-0. 56]	106	0.32 [0.20-0.44]	50	0.09 [0.05- 0.13]	68	0.11 [0.07-0.16]
Medical	586	1.59 [1.38-1.80]	262	0.59 [0.48-0.70]	351	0.84 [0.70-0.98]	83	0.13 [0.08-0.17]	63	0.15 [0.10-0.21]
Intensive care	151	3.08 [2.38-3.78]	84	1.44 [0.98-1.90]	49	0.85 [0.49-1.22]	74	0.98 [0.57-1.39]	39	0.60 [0.30-0.90]
Total	1166	1.64 [1.46-1.82]	523	0.75 [0.60-0.89]	506	0.74 [0.60-0.88]	207	0.25 [0.13-0.37]	170	0.22 [0.15-0.29]

* 95 % confidence interval

### Prevalence and origin of Gram-positive MDROs

**Fig. 2:**
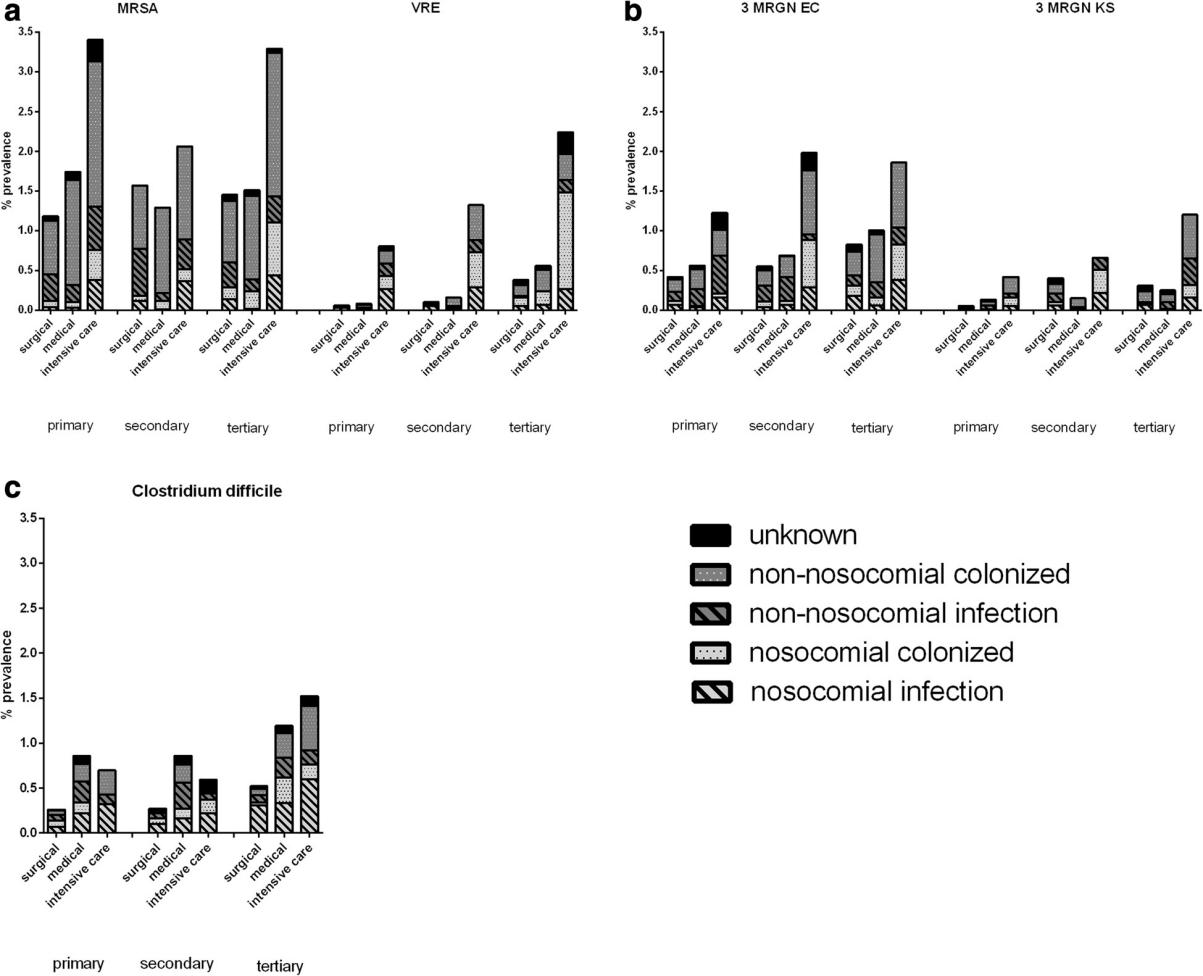
Prevalence, clinical presentation (infection or colonisation) and origin (nosocomial or non-nosocomial) of most reported MDRO cases (**a**: MRSA and VRE, **b**: 3MRGN EC and 3MRGN KS) and **c**: *Clostridium difficile*

Prevalence, clinical presentation (infection or colonisation) and origin (nosocomial or non-nosocomial) of most reported MDRO cases are shown in Fig. 2.

MRSA had a marked prevalence in all levels of care and types of wards (Fig. 2a). Most MRSA cases were colonisations of non-nosocomial origin (65.0 %). While the prevalence in surgical and medical wards was quite comparable between levels of care (ranging between 1.18 (surgical, primary level) and 1.78 % (medical, second level), ICUs had a much higher prevalence in all settings, ranging between 2.05 % (second level) and 3.39 % (primary level). While there was a trend to a higher MRSA-prevalence in hospitals that did screen for MRSA and hospitals that reported to have a universal screening policy reported the highest prevalence, confidence intervals did broadly overlap (Table 3). VRSA was almost absent in this survey. Only one case of nosocomial colonization from a tertiary level ICU was reported.

For VRE in contrast, there was a marked difference in the mean prevalence between levels of care with a trend to a higher prevalence in second and third level hospitals (Fig. 2a). Again, the prevalence in ICUs was much higher (ranging between 0.81 % and 2.25) compared to the other wards regardless of the level of care, making VRE the fourth most prevalent pathogen in this survey. As for MRSA, most cases were classified as colonisations but, the epidemiology was strongly determined by nosocomial origin. This became particularly evident for ICUs were 53.33 % (primary level) to 65.85 % (tertiary level) of cases were reported to be nosocomial. While there was a trend for a higher prevalence in hospitals that did screen for VRE, confidence intervals did overlap (Table 3).

### Prevalence of Gram-negative MDROs

Prevalence, clinical presentation (infection or colonisation) and origin (nosocomial or non-nosocomial) of the most prevalent Gram-negative MDROs 3MRGN-EC and 3MRGN-KS are shown in Fig. 2b. Again, ICUs had the highest prevalence. For 3MRGN-EC in particular there was a trend to higher prevalence in higher level of care hospitals.

The prevalence of 3MRGN-PS was only slightly lower than that of 3MRGN-KS (0.21 % [CI95: 0.10-0.31]) followed by 3MRGN-EB (0.10 % [CI95: 0.05-0.15], other not otherwise specified 3MRGN-organisms (0.10 % [CI95:0.05-0.15] and 3MRGN-AB (0.02 % [CI95:0.00-0.03]).

Besides 3MRGN-MDROs, 4MRGN-MDROs were reported as well. With a prevalence of 0.17 % [CI95:0.06-0.29] 4MRGN-PS was the seventh most common Gram-negative MDRO. Moreover, this organism was reported from all levels of care in a frequency that was comparable to 3MRGN-PS. While the prevalence of 4 MRGN-KS was low (0.04 % [0.00-0.07]), at least one case was reported from all levels of care and type of ward. The prevalence of other 4MRGN ranged between 0.01 % and 0.04 %.

With a prevalence of 0.74 % [CI95: 0.60–0.88] *Clostridium difficile* was the third most often reported pathogen in this survey (Table 5). Nosocomial cases made up a relevant proportion in all settings, as 31.17 % (medical wards, secondary level) to 65.22 % (surgical wards, primary level) of cases were of nosocomial origin (Fig. 2c). Still, most cases occurred on normal wards and did not require ICU-treatment. Less than 3 % (15 cases) were reported as severe cases (prevalence 0.04 % [CI95: 0.01-0.08]).

Hospitals that reported to have an ESBL-screening policy showed a significantly higher prevalence of 3MRGN-EC compared to hospitals that did not screen (*p* < 0.001) and a trend to higher prevalence rates for 3MRGN-KS, 3MRGN-EB and 3MRGN-PA (Table 3). Likewise, hospitals that screened for 4MRGN reported trend to higher prevalence rates of 4MRGN-KS, 3MRGN-EB, 3MRGN-AB and 3MRGN-PS. While the confidence intervals for 3MRGN-KS did not overlap, the difference was not statistically significant (*p* = 0.068).

## Discussion

Bacterial resistance has become one of the major challenges in the therapy and control of nosocomial infections. Studies assessing the prevalence of MDROs can help to improve the understanding of the epidemiology of MDROs as well as increase the awareness to this problem and thus foster the development and implementation of evidence based strategies to combat bacterial resistance.

Our study is the third survey in a row to assess the emergence of MDROs and *Clostridium difficile* as well as staffing with infection control personnel and MDRO screening policy in German hospitals. As in the previous surveys, we used a pragmatic, easily accessible approach by collecting routine surveillance data that has to be present in hospitals by law in Germany. By limiting the prevalence survey to intensive care units, surgical and medical wards, our data allows comparisons between primary, secondary and tertiary level hospitals. Furthermore, as this survey uses the same method as the previous surveys, temporal comparisons are possible in principle, too. When comparing the three surveys, the most obvious result is the steep increase in participating hospitals that grow from 9 hospitals in 2010 to 62 hospitals in 2012 and 364 hospitals in this survey. The increase from the first to the second survey can be explained by the support from the Action Group Infection Prevention (“Initiative Infektionsschutz”) (AGIP) and Ipse communication GmbH that provided an extensive list of e-mail contacts of hospitals. The 4-fold increase in the return rate from the former to this survey however can be interpreted as an expression of the growing awareness to MDROs and public acceptance of our easily accessible approach that depicts the “true” clinical situation as seen by health care workers and infection control personnel in the hospitals.

With the amendment of the German Protection against Infection Act in 2011, the recommendations from the German national committee for infection prevention Commission on Hospital Hygiene and Infection Prevention (KRINKO), the German national committee for infection prevention, became obligatory for hospitals and other health care facilities. As a consequence, staffing with infection control personnel became mandatory. The recommendations for staffing by the KRINKO are based on a risk assessment that take into account the treatment range of the medical provider as well as the individual risk profile of the treated patients [18]. However, a recent report of the federal government on nosocomial infections and bacterial resistance saw great variations in the compliance with this standard [20]. Due to the requirements for staffing with infection control consultants and infection control nurses, 82.2 % - 85.6 % (median: 84.8 %) and 69.7 % - 80.0 % (median: 74.8 %) of hospitals met the KRINIKO-recommendation, respectively [18, 20]. While still not perfect, our survey shows a good compliance with the recommendations as 84.8 % of hospitals met the staffing needs for infection control nurses and 74.8 % for infection control consultants. This again points to the assumption, that appropriate staffing with infection control personnel fosters hospital epidemiology and awareness and countermeasures against bacterial resistance.

Our data implies that there is still a relevant number of unknown MDRO cases, as the reported prevalence rates of most MDROs tends to be higher in hospitals that screen for particular MDROs than in hospitals that do not. While this problem may be negligible for MRSA as most hospitals reported to have some kind of screening in place, it may be influential for VRE and Gram-negative MDROs, as the majority of hospitals still reported to have no active screening policy.

Direct comparisons between prevalence rates reported in this survey and other surveys, including our former studies, should be made with caution as the samples are not identical and it is therefore unclear whether the same hospitals have participated. Still, comparisons between point prevalence studies have been made regardless of these problems [21–23]. Assured by the large number of participating hospitals in our study we will therefore discuss some general comparisons between our study and other studies to give an impression how our data fits to other surveys. The German part of the European point prevalence study of the European Centers for Disease Control (ECDC-PPS) from 2011 has been published as public report by the National Reference Center for Surveillance of Nosocomial Infections [21]. While the European point prevalence study dates back from 2011, the prevalence of nosocomial infections due to MRSA (ECDC-PPS (Germany): 0.17 %, our survey: 0.17 %), CD (ECDC-PPS (Germany): 0.30 %, our survey: 0.26 % and 3MRGN-EC (ECDC-PPS (Germany): 0.15 %, our survey: 0.12 %) fit well to our data, while the prevalence of nosocomial infections due to VRE is much higher in our survey (0.12 %) compared to the data from the German ECDC-PPS (0.02 %). This would fit to the increase in the VRE prevalence as recently reported by the National Reference Center for Surveillance of Nosocomial Infections [24].

A direct comparison to prevalence rates reported by the German Surveillance System of Nosocomial Infections in Hospitals (KISS) on the other hand is not possible, as this system reports incidences and prevalence rates of particular of some MDROs but uses an entirely different way of data collection and statistics [22, 25]. For example, MRSA-KISS reports MRSA prevalence rates and incidences on a yearly base, but does not separate by medical specialties, infections and colonizations. Moreover, prevalence rates and incidences are not calculated per hospital but pooled over all participating hospitals and over the whole time [26]. Other KISS modules (ITS/Stations-KISS) on the other hand does report prevalence rates and incidences divided by medical specialties, infections and colonizations but not on a yearly base but pooled from 2013 [27].

Our results underlay that prevalence data reported by KISS cannot be used as valid indicators for the daily prevalence of MDRO patients in hospitals. This further explains why the situation felt by health care workers and infection control personnel in the hospitals does not fit well to the reported prevalence data, as the perceived epidemiological situation is mainly determinated “burden”.

Compared towards our former survey from 2012, MRSA, VRE, 3MRGN-EC, 3MRGN-KS and CD remain the most prevalent pathogens in all levels of care and types of wards (Table 7) [12]. The prevalence of MRSA and VRE almost matches the ones from the former survey. Therefore MRSA still remains the most prevalent and most prominent MDRO in German hospitals. The prevalence of 3MRGN-EC and 3MRGN-KS are both somewhat lower compared to the prevalence of ESBL-EC and ESBL-KS reported in 2012, but confidence intervals broadly overlap. The trend to a lower prevalence can be explained by the stricter definition for 3MRGN is than for ESBL. Only CD infections had an obviously lower prevalence in comparison to 2012. An in depth comparison showed that especially the prevalence on surgical and internal ward was lower compared to 2012 (with confidence intervals non overlapping), while the confidence intervals did broadly overlap for ICUs. Whether this indicates a true reduction in CD cases (e.g. caused by better antibiotic stewardship), shorter duration of stay of CD cases (reducing the chance to be detected in a point prevalence study) or other factors, remains unclear. Either way, CD still remains one of the most prevalent pathogens.

**Table 7:** Data of mean prevalence of the most prevalent MDROs of point prevalence survey 2012 [12] and 2014

Year of point prevalence study	MRSA		VRE		ESBL-EC (2012) 3MRGN-EC (2014)		ESBL-KS (2012) 3MRGN-KP* (2014)		CD	
	Mean prevalence [95 % CI]		Mean prevalence [95 % CI]		Mean prevalence [95 % CI]		Mean prevalence [95 % CI]		Mean prevalence [95 % CI]	
2012 [12]	1.53	[1.32 – 1.75]	0.27	[0.18 – 0.36]	0.97	[0.80 – 1.14]	0.33	[0.19 – 0.48]	1.30	[1.11 – 1.50]
2014	1.64	[1.46 – 1.82]	0.25	[0.13 – 0.37]	0.75	[0.60 – 0.89]	0.22	[0.15 – 0.29]	0.74	[0.60 – 0.88]

* Klebsiella pneumonae

Our study has several limitations that should be acknowledged when interpreting the data. First, as we used routine data, our results have the typical constrains associated with this type of data. We deliberately gave no extra definition for cases, or to distinguish between infections/colonisations, nosocomial and hospital acquired cases. This is a complementary approach to other epidemiological studies on the same topic that use generic definitions (e.g. CDC-definitions) to classify cases.

The obvious drawback of our method is that there may be some differences in the definitions between hospitals or even wards. However, our approach has the advantage that our results depict the “true” clinical situation as seen by health care workers and infection control personnel in the hospitals. Moreover, this data are used for calculating of reimbursement by health insurances, too.

Second, as the survey was voluntary and anonymous, we have no means to validate the results and our sample may not be representative. Still, with almost 74,000 patients from 329 hospitals, our survey uses data from more than 16 % of German hospitals. It is therefore the largest study of its kind in Germany with more than twice as many participating hospitals as the in the German part of the ECDC point prevalence study from 2011 [21]. This underlies that pragmatic surveys to collect routine surveillance data are an attractive and replicable way to gather epidemiological data with limited resources. Voluntariness and self-reporting are an issue for other surveys, like the German hospital infection surveillance system (KISS), too. As in the previous surveys, data was collected directly at the wards or using electronic systems by trained personnel in most hospitals. Therefore, we are quite confident that our results give a good estimate of the situation in German hospitals.

Our results should be used by infection control personal to raise awareness towards bacterial resistance and foster infection control measures. Further studies should follow up our results and expand to other health care settings as rehabilitation and nursing homes.

## Conclusion

Point prevalence surveys that use a straight forward approach allow making routine data available for epidemiological research and can help to raise awareness about bacterial multi-resistance. Our study indicates that MRSA, 3MRGN-EC, CD, VRE and 3MRGN-KS remain the most prevalent pathogens in all levels of care and types of wards in Germany. While most cases are reported to be non-nosocomial, nosocomial transmission and infection plays a significant role especially on ICUs that also show the highest prevalence rates of MDROs.

## Abbreviations

AB, *Acinetobacter spp.* ; AGIP, Action Group Infection Prevention; CD, *Clostridium difficile*; EC, *Escherichia coli*; ECDC, European Centers for Disease Control; ES, *Enterobacter spp.*; ESBL, extended-spectrum beta lactamase; HICARE, Health, Innovative Care and Regional Economy; ICC, infection control consultant; ICN, infection control nurse; ICU, intensive care unit; KISS, German hospital infection surveillance system; KRINKO, German national committee for infection prevention; KS, *Klebsiella spp.*; LPIC, link physician for infection control; MDROs, multidrug resistant bacterial organisms; MRGN, multidrug resistant Gram-negative; MRSA , Methicillin-resistant *Staphylococcus aureus*; PPS, point prevalence study; PS, *Pseudomonas spp.*; QM, quality management; spp., species pluralis; VRE, Vancomycin resistant *Enterococcus faecalis/faecium;* VRSA/GRSA, Vancomycin resistant *Staphylococcus aureus*
